# Silver linings: a personal memoir about Hurricane Katrina and fungal volatiles

**DOI:** 10.3389/fmicb.2015.00206

**Published:** 2015-03-18

**Authors:** Joan W. Bennett

**Affiliations:** Department of Plant Biology and Plant Pathology, School of Environmental and Biological Sciences, Rutgers – The State University of New Jersey, New Brunswick, NJ, USA

**Keywords:** volatile organic compounds (VOCs), sick building syndrome, 1-octen-3-ol (mushroom alcohol), double petri plate system, *Aspergillus*

## Abstract

In the aftermath of Hurricane Katrina, the levees protecting New Orleans, Louisiana failed. Because approximately 80% of the city was under sea level, widespread flooding ensued. As a resident of New Orleans who had evacuated before the storm and a life-long researcher on filamentous fungi, I had known what to expect. After the hurricane I traveled home with a suitcase full of Petri dishes and sampling equipment so as to study the fungi that were “eating my house.” Not only were surfaces covered with fungal growth, the air itself was full of concentrated mold odor, a smell that was orders of magnitude more funky than any damp, musty basement I had ever encountered. The smell made me feel bad and I had to take regular breaks as I sampled. Being a mycotoxin expert, I knew a fair amount about “sick building syndrome” but believed that it was difficult to get enough respiratory exposure to toxins to cause the array of symptoms associated with the syndrome. So why was I feeling sick? Some Scandinavian experts had hypothesized that mold volatile organic compounds (VOCs) might be the fungal metabolites to blame for sick building syndrome and the time in my smelly, mold infested home made me think they might be right. After securing a new job and establishing a new laboratory, I endeavored to test the hypothesis that some volatile mold metabolites might be toxic. My laboratory at Rutgers University has interrogated the role of VOCs in possible interkingdom toxicity by developing controlled microcosms for exposing simple genetic model organisms to the vapor phase of growing fungi. Both *Arabidopsis thaliana* and *Drosophila melanogaster* exhibit a range of toxic symptoms that vary with the species of fungus, the duration of exposure, and other experimental parameters. Moreover, low concentrations of chemical standards of individual fungal VOCs such as 1-octen-3-ol also exhibit varying toxicity and cause neurotoxicity in a *Drosophila* model. Collectively, these data suggest that fungal VOCs may contribute to some of the adverse health effects reported by people exposed to damp indoor environments and that biogenic gas phase molecules deserve increased attention by the research community.

## Introduction

I am a fungal geneticist. For most of my professional career, I lived in New Orleans, Louisiana, USA where I was on the faculty at Tulane University and maintained a strong collaboration with scientists at the Southern Regional Research Laboratory, a branch of the U. S. Agricultural Research Service. My research concerned the genetics and physiology of fungal toxins (mycotoxins), especially the aflatoxins produced by *Aspergillus flavus* and *Aspergillus parasiticus*. I had plenty of experience studying filamentous fungi and was a self-proclaimed mycophile. Nevertheless, I never expected that fungi would change my life outside of the laboratory. Yet, that is pretty much what happened to me late in the summer of 2005, in the aftermath of Hurricane Katrina. Here is my story.

## A Hurricane Memoir

In August 2005 I was about to start a sabbatical leave during which I planned to work on the annotation of the genome of *Aspergillus flavus*, an aflatoxigenic species that had just been sequenced with funding by the U. S. Department of Agriculture. My sabbatical plans were permanently altered on August 29, 2005 when Hurricane Katrina crossed the Gulf of Mexico. The day before the hurricane, my husband and I evacuated to a small town in eastern Louisiana so we were not in New Orleans when the hurricane hit and the levees failed. About 80% of the city flooded, including our house. The National Guard barred residents from returning home. Therefore, we drove to New Jersey, where my husband had friends, and found temporary housing. The weeks after Hurricane Katrina were not easy. At first, I watched a lot of TV, getting increasingly angry at the negative media slant put on New Orleans and its residents. A retired colleague, Dr. Gerhard Haas, asked me to give a lecture about my hurricane experiences at Fairleigh Dickinson University, so I used the internet connection at the Franklin Lakes, NJ, public library to obtain images, learn more about the devastation, and read up on categorizations used for hurricanes (1–5). I learned that although Katrina originally had been a powerful Category 5 storm, by the time it hit New Orleans it was “only a high 3.” Nevertheless, storm surges caused by the hurricane breached the levees protecting the city and inundated areas below sea level. It took several weeks for the Army Corps of Engineers to pump the flood water out of the city. During that time, public health officials expressed concerns about infectious disease, water pollution, and heavy metal contamination. Almost no one paid any attention to mold. But I knew that the city – the drowned and dying vegetation, the water logged buildings, and their contents – were perfect substrates for filamentous fungi. New Orleans had become a paradise for molds and mildews.

My husband I did not return to New Orleans until early October. Good friends whose home had not flooded invited us to stay in their guest room. Before leaving New Jersey, I called a professional acquaintance, Dr. James White, Chair of the Plant Biology and Pathology, Department at Rutgers University and asked if I could come to his lab and make media. He readily agreed. So on October 4th, 2005 when my husband and I flew back to New Orleans for the first time since our evacuation, I traveled with a suitcase containing sleeves of sterile Petri plates containing fungal media, sampling equipment, disposable latex gloves, and face masks.

**FIGURE 1:**
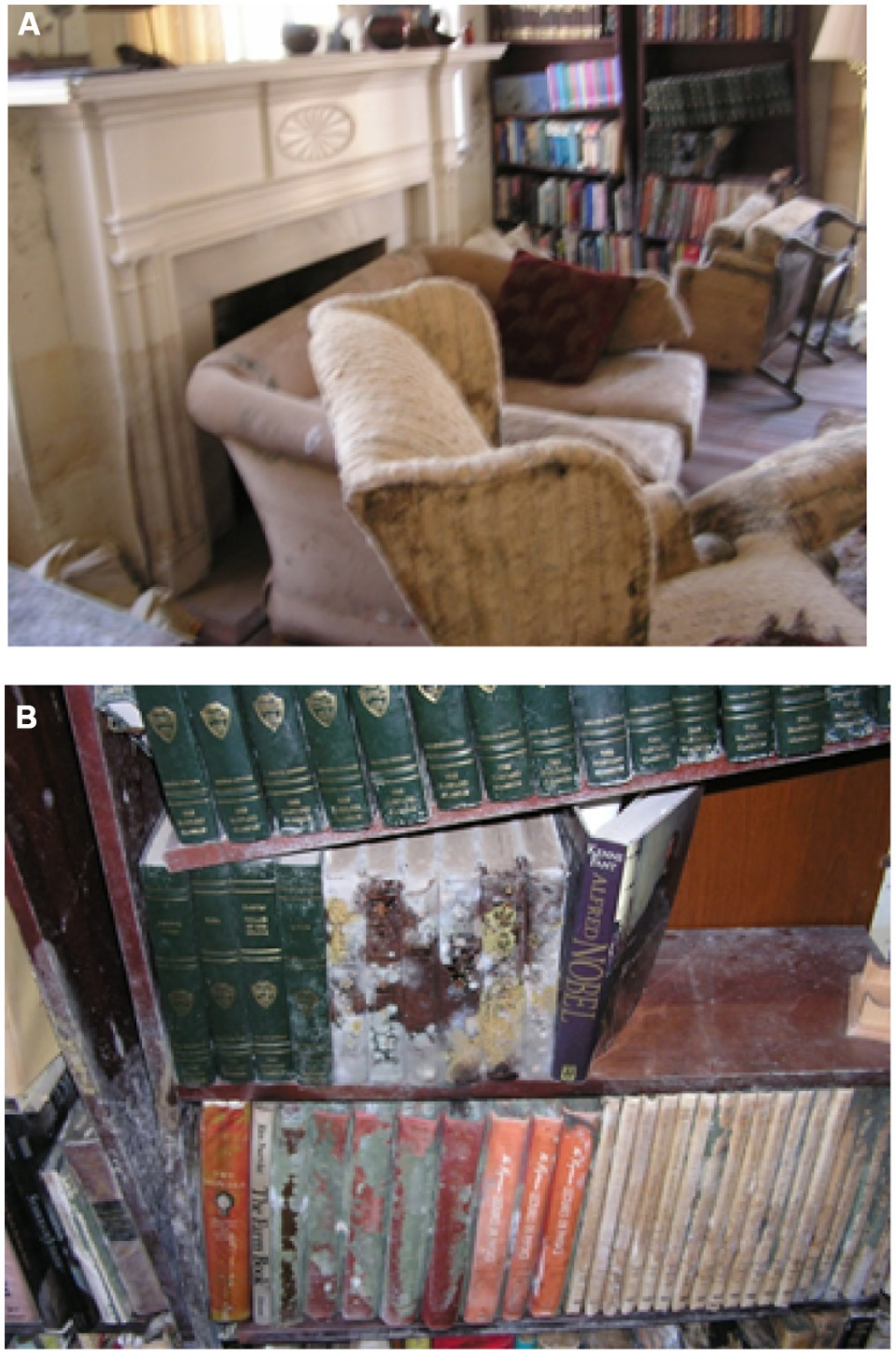
**(A)** The living room of the author’s New Orleans home on October 6, 2005 in the aftermath of Hurricane Katrina. **(B)** Closer view of moldy books on flooded book shelf.

Before evacuating New Orleans, we had shut the windows, pulled the curtains, and locked all the doors to our house. We also had taken a last look at our beautiful green garden. By the time we returned to our neighborhood, the flood water was gone but most of the vegetation was dead from the prolonged anoxia. The lawns and our half-century-old azalea bushes were brown and shriveled. My husband and I did our best to brace ourselves. Nevertheless, when we opened the front door to our home, the reality of the devastation was hard to take (see **Figures 1A,B**).

There was no longer any standing water in the house but the carpets were still squishy; a car we had left behind in the garage was filled with muddy water. Several armchairs were lying on their side. We surmised that when the flood waters surged in they had knocked over the lighter pieces of furniture. As expected, mold was growing on almost every surface – carpets, curtains, upholstered furniture, and most heart breaking of all, some of my most beloved books. Our home had become a fungal utopia. What was unexpected was the intensity of the stench. Like everyone, I was familiar with the smell of damp basements, attics, and other spaces where microbial life grows in enclosed spaces. As an *Aspergillus* geneticist, I also was familiar with the characteristic scent that hits you when you open the door of an incubator full of growing fungi. But nothing had prepared me for the intensity of the odor in our flooded home. The stench was so strong that it didn’t really smell like mold – it just smelled horribly funky and powerfully sickening.

For me, being able to slip into my role as a professional scientist helped with the psychological shock. I proceeded to sample the house. As usual, it was very hot in New Orleans and the lack of electric power meant there were no fans, much less any air conditioning. The combination of the heat, the emotional impact of seeing my mold-ravaged home, and the terrible smell of rot in the enclosed rooms made me feel sick. Since I was wearing a mask while I sampled, I didn’t think that my physical reaction came from breathing mold spores. Nevertheless, my body was telling me, “Get out of here.” So, I wiped off one of the wooden chairs and put it in the front yard. Over the next few hours I alternated my mold sampling and photography with sitting in the front yard, breathing the outdoor air, and letting my mind wander. Among other things, I thought about the bad smell and wondered where it came from. Were the odors caused by mold metabolism or were they incidental breakdown products of fungal degradative enzymes? I knew that fungi made odorant metabolites but I had never given them much attention. For example, I knew that some mushroom hunters use characteristic scents to help them identify delectable species. Chanterelles are known for their fruity, apricot-like odor, and the princess matsutake *Agaricus subrufescens* smells like almonds (Harper et al., 1968; Cronin and Ward, 1971). Further, mold volatiles have been used to detect contamination in stored grains (Schnürer et al., 1999). I also had read that workers in Scandinavia had postulated that mold volatile organic compounds (VOCs) might be responsible for some of the symptoms associated with “sick building syndrome,” a poorly defined condition of unknown etiology that was associated with unhealthy indoor environments. With the benefit of hindsight, I wish that I had brought appropriate equipment for sampling VOCs but lacking such technological supports, I nevertheless started to form my own conjectures. Perhaps my own negative physical reaction was caused by the fungal odorants. Perhaps some mold VOCs were biologically active. Perhaps if I ever could re-establish my laboratory, I could devise a way of studying the toxigenic potential of fungal VOCs.

## Starting Over

**FIGURE 2:**
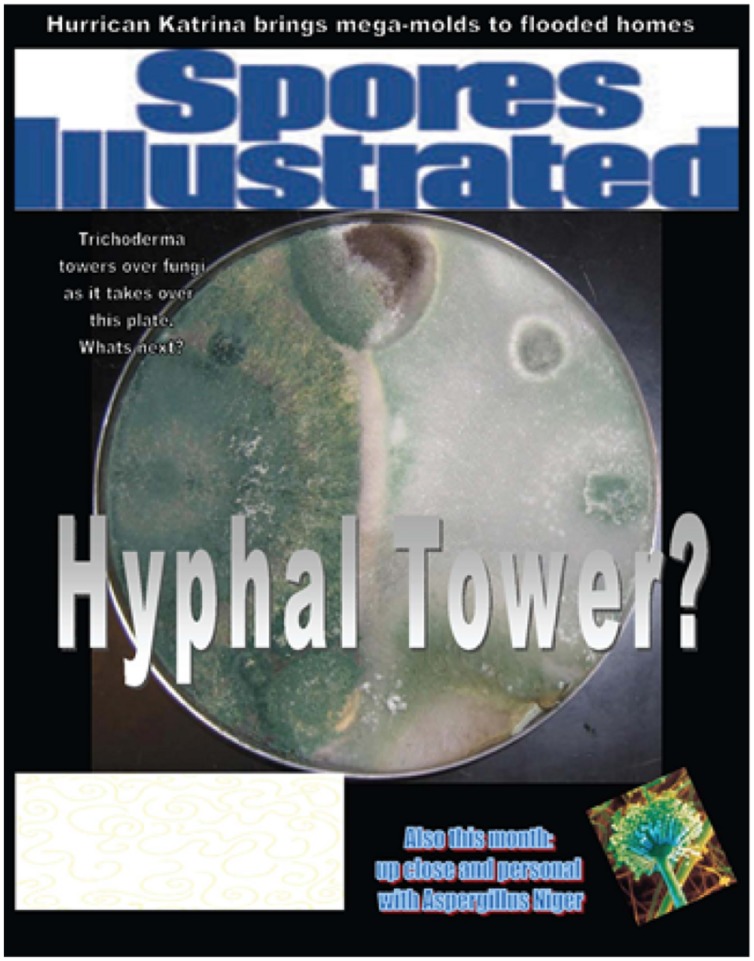
**Cover of undergraduate research paper about molds isolated after Hurricane Katrina, designed by Craig Pritch, May 2006**.

Now to flash forward and abbreviate several difficult months. My husband and I hired a crew of workers to gut our house and then we flew back to New Jersey. I brought my Katrina molds to Rutgers and subcultured them. Jim White suggested that I should meet with Robert Goodman, Dean of Cook College (now School of Environmental and Biological Sciences), the agricultural unit of Rutgers University. Dean Goodman invited me to complete my sabbatical at Rutgers. The following January we rented a house near the Rutgers campus and I started a new research project. I worked with a an undergraduate named Craig Pritch doing tentative taxonomic identifications of my Hurricane Katrina molds and reading the literature on sick building syndrome. At the end of the semester, Mr. Pritch submitted a short research paper with a humorous cover page showing a contaminated culture of *Trichoderma*, one of the most common molds found in my flooded home (see **Figure 2**). Despite the spelling errors, it remains my favorite-ever cover sheet for a student paper.

During my sabbatical time in the Department of Plant Biology and Pathology, Dr. Philip Furmanski, Executive Vice President of Rutgers University offered me a permanent job at the university and I accepted. The day after I received my tenure letter from Rutgers University I resigned from Tulane University. Although I was a senior scientist, I had decided that I would start a new research career, studying the possible physiological activity of fungal odorants. My husband I moved permanently to New Jersey in late August 2006, almost exactly 1 year after the hurricane that had changed our lives.

**FIGURE 3:**
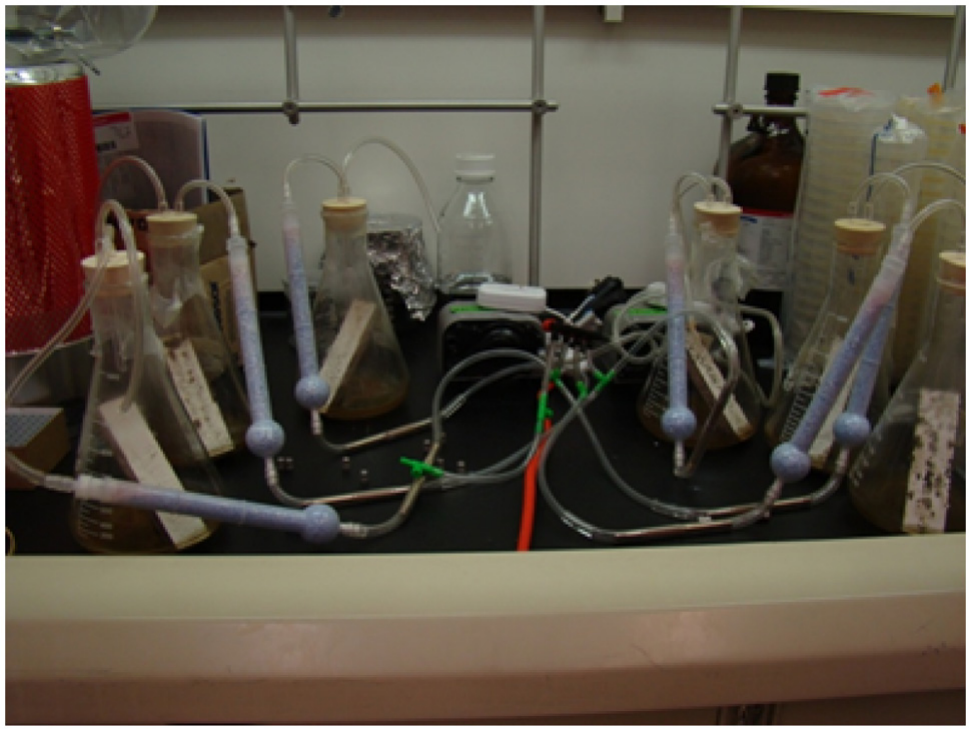
**Experimental set up for collecting volatiles emitted by molds growing on gypsum board (Image courtesy of Richard Hung)**.

During my early months on the Rutgers faculty, I recruited my first new graduate student, Richard Hung, who had worked in my lab as an undergraduate at Tulane, and my first visiting scientist, Prakash Masurekar who had recently retired from Merck Corporation. They helped order supplies, equipment and set up the lab. We put the Katrina molds that I had collected into pure culture and then grew them individually on laboratory medium and gypsum board (“sheet rock”) in controlled microcosms. Richard Hung spent rather a lot of time working on developing appropriate collection methods (See **Figure 3**).

The fungal isolates were grown on gypsum board, VOCs from the head space collected, and using gas chromatography–mass spectrometry (GC–MS), profiles of VOCs were identified. The VOCs consisted of mixtures of different chemical classes: alcohols, aldehydes, hydrocarbons, aromatics, nitrogen-containing compounds, thiols, terpenoids, and their derivatives. Previous researchers had done a good job of cataloging the VOC profiles produced by “indoor molds” grown on different substrates and similar profiles of metabolites previously had been identified from contaminated building materials (Fiedler et al., 2001; Claeson et al., 2002; Matysik et al., 2008). Thus, we learned what others had discovered and published before us. Molds make many different VOCs. Different species of molds make different combinations of VOCs. They make them in different proportions and concentrations, depending on how long they are grown, and what they are grown on. These VOCs are produced as blends of complex mixtures that change dynamically with time.

## Some Background on Sick Building Syndrome

For decades the international public health community has been concerned about the possible adverse health effects of molds in damp indoor environments. These adverse health effects are usually named “sick building syndrome” or less frequently “damp building syndrome” (Godish, 1995; Hodgson, 2000). Sick building syndrome is characterized by a group of non-specific symptoms that include fatigue, respiratory distress, skin problems, eye irritation, mental disturbances, and so forth (Hodgson, 2000, 2002; Burge, 2004). The “off gassing” of industrial solvents, air borne particulates, and exposure to mold toxins (mycotoxins) have been implicated as causes of this difficult-to-define syndrome (Godish, 1995; Straus, 2009) with molds considered one of the main risk factors (Li and Yang, 2004). Some experts question whether there is sufficient evidence to link molds and mycotoxins to the wide range of symptoms associated with this elusive health condition (Hardin et al., 2003; Kuhn and Ghannoum, 2003). Both the U. S. Institute of Medicine [IOM], 2004 and the World Health Organization [WHO], 2009 have published meta-reviews of the published literature in which it was concluded that certain respiratory health effects (e.g., asthma, allergy, and upper respiratory tract irritation) show an association with mold exposure but that there is no conclusive evidence linking mold exposure to the full set of symptoms attributed to sick building syndrome. Many data gaps exist. Available studies have shown a lack of standardized methods for measuring exposure to molds and difficulty in determining which of several disease-causing agents in damp indoor environments may be responsible for the adverse health effects (Institute of Medicine [IOM], 2004; World Health Organization [WHO], 2009). Moreover, although there is a large published literature on the adverse health effects of mycotoxins, far less is known about fungal VOCs. Danish workers were the first to postulate that VOCs caused or contributed to the adverse health effects associated with unhealthy indoor environments (Mølhave, 1992; Mølhave et al., 1993). Several laboratories have shown that certain industrial and biogenic VOCs are toxic in mammalian cell culture (Kreja and Seidel, 2002; Korpi et al., 2009). Furthermore, human volunteers exposed to vapors of mushroom alcohol (1-octen-3-ol) at a concentration of 1.9 ppm for 2 hours show an increase in inflammatory markers in their nasal secretions (Wålinder et al., 2008).

Obviously, people who complain of building-related ill health may have been exposed to numerous toxigenic and allergenic agents. These include interacting biological (e.g., mycotoxins), chemical (e.g., off-gassing of building materials), and physical (e.g., ventilation rates) factors that may interact synergistically to create “problem buildings.” For detailed discussions of the complexities involved in the analysis of sick building syndrome, including psycho-social dimensions, see the monographs by Cone and Hodgson (1989), Godish (1995), and Straus (2009).

## Developing Model Systems

After cataloging the VOCs produced by our Katrina molds, we embarked on a new avenue of research that to our knowledge has not been done by other groups. We pioneered the use of genetic model systems to test the physiological activity of fungal VOCs in controlled environments. Our intent was to create simplified systems in which to isolate individual factors and to do so in model organisms that would allow us to investigate the mechanistic basis of any effects we uncovered. A long term goal was to create standardized toxicological assays for studying fungal volatile compounds in hopes of finding a way to link mold VOCs and negative health consequences.

### Caenorhabditis elegans

Richard Hung obtained several strains of *Caenorhabditis elegans* and cultured them in the presence of growing molds or low concentrations of chemical standards of several of the VOCs we knew were components of the blends emitted by growing molds. We envisioned that we would record the behavior of wild type worms and then test mutants lacking certain known chemosensory genes. However, the experiments did not go well. After a short time, many worms would be found having crawled up the sides of the Petri plates. Most of the nematodes seemed to have vanished. We speculated that they crawled out of the plates and desiccated. Because these nematodes are tiny and translucent, we could not even find their dead bodies. We abandoned *C. elegans*.

### Arabidopsis thaliana

Based on the observation that vegetative growth is suppressed in areas known to have truﬄes growing underground, Italian workers had hypothesized that plants may have the ability to detect the presence of fungi through their volatiles (Splivallo et al., 2007). The same workers determined that at high concentrations, the distinctive fungal VOC commonly called “mushroom alcohol” (1-octen-3-ol) inhibited root growth and lowered chlorophyll concentration in *Arabidopsis thaliana*. A Japanese group had shown that at lower doses, 1-octen-3-ol enhanced resistance of mature *A. thaliana* to *Botrytis cinerea*, and activated some of the same defense genes turned on by ethylene and jasmonic acid signaling (Kishimoto et al., 2007). Therefore, Richard switched his attention to using *A. thaliana* as a possible genetic model for assaying volatile toxicity. He determined that a low concentration (1 ppm) of racemic, (S)-(+)-1-ocen-3-ol or R–(-)-1-octen-3-ol had negative effects on *A. thaliana* seedling formation, biomass production, chlorophyll content, and electrolyte leakage of 2 week old plants (Hung et al., 2014a). Further, Richard joined with Samantha Lee, another graduate student in the lab, and evaluated the sensitivity of *A. thaliana* to the presence of eight biogenic and six anthropogenic VOCs using seed germination and plant growth assays. With the exception of ethanol, when compared to controls, all the VOCs we studied gave rise to significantly lower levels of chlorophyll in treated plants. Many showed severe discoloration and curling of leaves as well as localized cell death (Lee et al., 2014). Then, in separate experiments when testing the effect of exposing *A. thaliana* to VOCs from growing fungi, Richard unexpectedly discovered that the VOCs from a *Trichoderma* species *enhanced* plant growth (Hung et al., 2013, 2014b). *Trichoderma* is well known and widely used as a biocontrol species (Harman et al., 2004) but the contribution of its VOCs to plant vigor had not before been shown. Therefore, our laboratory has established a new research project that focuses on the plant growth enhancement effects of *Trichoderma* VOCs and shown that the effects are not limited to *A. thaliana* (unpublished data).

### Drosophila melanogaster

**FIGURE 4:**
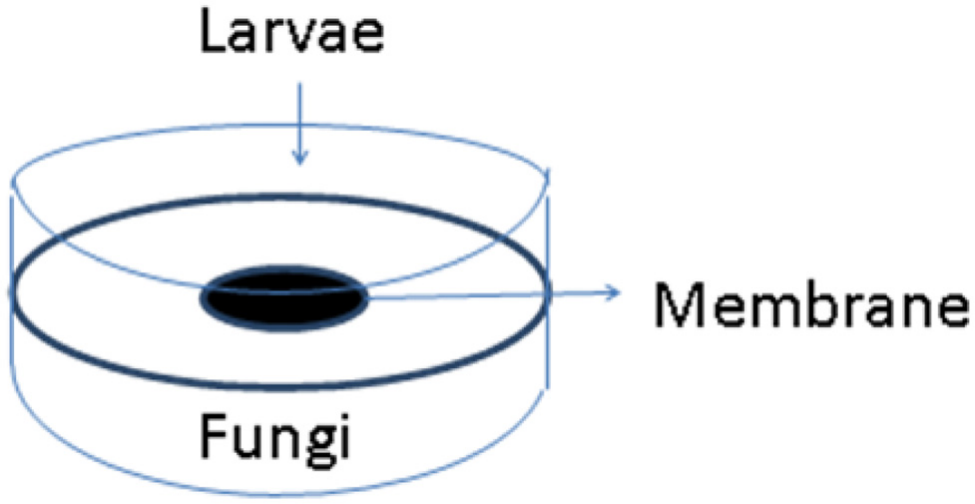
**Double Petri plate system to test volatile organic compounds (VOCs) emitted from a living culture of mold**. *Drosophila* third instar larvae are placed on one plate and a growing mold culture on the other. A membrane permeable to VOCs separates the two plates (Image courtesy of Arati Inamdar).

About 2 years after the Hurricane, I hired a postdoctoral research associate named Arati Inamdar who had training in neurobiology. She developed a new bioassay using *Drosophila melanogaster*. We exposed both adult flies and third instar larvae. For the latter assay Arati devised double petri plate “sandwiches” consisting of two Petri dish bottoms separated by a lid into which a hole had been burned (see **Figure 4**). The hole was sealed carefully with a membrane that allowed VOCs to pass. On one side, we placed the fly larvae. In the other, we inoculated a growing culture of a mold isolated from my flooded home. We sealed the two plates together with Parafilm, placed them on a rotary shaker at constant temperature, and recorded the number of larvae, pupae, and adults that formed over a 2 week period.

The developmental stages of the flies were exposed to the VOC blends emitted by the growing fungi for the entire time. Isolates of *Aspergillus*, *Penicillium*, and *Trichoderma* from my flooded home were tested. Of these, the *Trichoderma* isolates were most toxic (Inamdar et al., 2012). Although different toxigenic effects were detected, it was impossible to know which specific compounds, or combination of compounds, were responsible for the negative effects we were seeing on fly metamorphosis and survival. The ever-changing profile of VOC production made it difficult to characterize biological effects. Which of the many VOCs was causing a given effect? Were multiple VOCs interacting in some synergist fashion? How much did substrate, and how much did genotype, dictate the kinds of VOCs produced?

After a long career as a geneticist, my mental preferences are reductionist. I don’t like experiments where there are too many variables acting simultaneously. Therefore, we decided to come at our hypothesis from a different direction. Rather than exposing our model to the ever-changing mixture of VOCs emitted by the growing molds, we turned to using chemical standards of individual VOCs. In this way, we could study them one by one using carefully calibrated concentrations. We went to a chemical supply catalog and looked to see which common fungal VOCs were available for purchase. We bought a number of the cheapest ones and then, for each chemical, we tested a range of low concentrations against both third instar larvae and adult flies. For positive controls, we tested the vapor phase of toluene, benzene, formaldehyde, and xylene. After 15 days of exposure, benzene and toluene led to 50% mortality while mortality was under 20% for formaldehyde and xylene. The percent mortality for 1-butanol, 1-propanol, 1-hexanol, and 1-decanol were 2, 42 and 68%, respectively. The eight carbon compounds we tested were more toxic to adult and larval *Drosophila* than the non-C8 compounds. Of these, 1-octen-3-ol (mushroom alcohol) and 3-octanol are secondary alcohols while 3-octanone is a ketone. All these C8 compounds yielded 100% mortality within 24 h (Inamdar et al., 2012). Further, the flies exposed to 1-octen-3-ol demonstrated a number of movement disorders, similar to those seen in a fly model for Parkinson’s disease (Inamdar et al., 2010). We went on and used the fly model, applying a combination of genetic, biochemical, and immunological approaches, to show that 1-octen-3-ol reduced dopamine levels and caused dopamine neuron degeneration in *D. melanogaster.* Furthermore, over-expression of the vesicular monoamine transporter (VMAT) rescued the dopamine toxicity and neurodegeneration. In contrast, mutations decreasing VMAT and tyrosine hydroxylase exacerbated toxicity. Uptake of dopamine was inhibited by vapor phase 1-octen-3-ol in human cell lines expressing the human plasma membrane dopamine transporter and human VMAT ortholog, VMAT2. Collectively, these data suggested that 1-octen-3-ol exerted toxicity via disruption of dopamine homeostasis and may represent a naturally occurring environmental agent involved in parkinsonism-like toxicity (Inamdar et al., 2013a). We also demonstrated that exposure of flies to 1-octen-3-ol stimulated the caspase-3 dependent apoptotic signaling pathway (Inamdar et al., 2013b). In addition, we showed that there was an induction of nitric oxide (NO) and its derivative, peroxynitrite by 0.5 ppm of 1-octen-3-ol an inflammatory response mediated via hemocytes, which are *Drosophila* innate immune cells. In other words, this ubiquitous fungal VOC, commonly associated with mold-contaminated damp indoor spaces, stimulated a NO mediated inflammatory response in nervous and respiratory tissues of *D. melanogaster* (Inamdar and Bennett, 2014). Together, these experiments with the *Drosophila* model may open a new avenue for mechanistic understanding of the possible human health effects of mold-emitted volatile chemicals.

## Conclusion

The toxicity and biological potency of a number of industrial solvents such as formaldehyde, toluene, and benzene are well known, however, far less is known about the gas-phase biomolecules secreted by fungi, bacteria, and green plants. Some of the most penetrating studies have been conducted by entomologists who have shown that many VOCs serve as semiochemicals (“infochemicals”; Davis et al., 2013). In general, the literature on biogenic VOCs is scattered between food and flavor chemistry, entomology, chemotaxonomy, and a number of other subdisciplines that do not normally “talk to one another.” Members of our laboratory, along with collaborators at Penn State in Seogchan Khan’s group, have collaborated on writing several review articles in an attempt to bring together the literature on VOCs that has been developed in mycology, entomology, building science as well as the elegant chemical studies performed by food and flavor scientists (Morath et al., 2012; Bennett et al., 2013; Bitas et al., 2013). Another interesting research development is the use of volatiles as a non-invasive method for disease detection. For example, the “volatome” of *Aspergillus fumigatus* has been analyzed and may provide an early diagnostic tool for systemic aspergillosis infections (Heddergott et al., 2014).

We believe that in order to bring the research on fungal volatiles to its appropriate place in 21^st^ century biology, we need better communication between fungal biologists, molecular biologists, entomologists, chemical ecologists, toxicologists, and all biologists interested in VOCs. The traditions of our respective disciplines do not make these associations easy to achieve, nor do the discipline-based approaches of the review committees associated with most funding agencies. The scientific community has a great deal to learn about the way in which VOCs influence ecosystem dynamics, especially the microbial ecosystems that function in indoor environments. The study of gas phase molecules produced by fungi increases our understanding of how fungi interact with their environment and with each other.

For me personally, the shift in my research focus has been intellectually stimulating. Sometimes bad experiences lead to good outcomes. That is what happened to me with Hurricane Katrina. The metaphoric black clouds of my Hurricane Katrina experience have provided a scientific silver lining and an entirely new research focus. The hurricane transformed my life. My flooded home, covered with mold growth, is what inspired my new research. I have changed jobs, the place where I live, and my perspectives about fungal metabolism. Like most biologists and biochemists, previously my experimental strategies were all based on “liquid phase biology.” Even when I studied water-insoluble mycotoxins, I grew molds in liquid media and then partitioned target metabolites into liquid-phase, non-polar solvents. During the many decades when I worked on aflatoxin genetics and biosynthesis, I cultured thousands of plates and flasks of fungi in laboratory incubators. When I opened an incubator door and smelled that characteristic moldy smell, I ignored it. Now my “consciousness has been raised.” I am alert to odors of all kinds – not just fungal odors – and have come to believe that volatile phase biology is a new scientific frontier. In nature, organisms do not live alone. They live in communities where interspecific communications frequently take place through gas-phase chemical signaling, especially in terrestrial environments. The possibilities for new discoveries are enormous. Scientists merely have to “open their noses,” smell the world around them, and recognize that odorants have many undiscovered biological properties. We hope that our exploratory research will inspire others to work on volatile phase signaling in biology.

## Conflict of Interest Statemen

The author declares that the research was conducted in the absence of any commercial or financial relationships that could be construed as a potential conflict of interest.
